# Molecular Footprints of the Immune Assault on Pancreatic Beta Cells in Type 1 Diabetes

**DOI:** 10.3389/fendo.2020.568446

**Published:** 2020-09-15

**Authors:** Maikel L. Colli, Florian Szymczak, Decio L. Eizirik

**Affiliations:** ^1^ULB Center for Diabetes Research, Medical Faculty, Université Libre de Bruxelles (ULB), Brussels, Belgium; ^2^Welbio, Medical Faculty, Université Libre de Bruxelles (ULB), Brussels, Belgium; ^3^Indiana Biosciences Research Institute, Indianapolis, IN, United States

**Keywords:** type 1 diabetes, beta cells, pancreatic islets, insulitis, inflammation, therapeutics, interferon, RNA-sequencing

## Abstract

Type 1 diabetes (T1D) is a chronic disease caused by the selective destruction of the insulin-producing pancreatic beta cells by infiltrating immune cells. We presently evaluated the transcriptomic signature observed in beta cells in early T1D and compared it with the signatures observed following *in vitro* exposure of human islets to inflammatory or metabolic stresses, with the aim of identifying “footprints” of the immune assault in the target beta cells. We detected similarities between the beta cell signatures induced by cytokines present at different moments of the disease, i.e., interferon-α (early disease) and interleukin-1β plus interferon-γ (later stages) and the beta cells from T1D patients, identifying biological process and signaling pathways activated during early and late stages of the disease. Among the first responses triggered on beta cells was an enrichment in antiviral responses, pattern recognition receptors activation, protein modification and MHC class I antigen presentation. During putative later stages of insulitis the processes were dominated by T-cell recruitment and activation and attempts of beta cells to defend themselves through the activation of anti-inflammatory pathways (i.e., IL10, IL4/13) and immune check-point proteins (i.e., PDL1 and HLA-E). Finally, we mined the beta cell signature in islets from T1D patients using the Connectivity Map, a large database of chemical compounds/drugs, and identified interesting candidates to potentially revert the effects of insulitis on beta cells.

## Introduction

Pancreatic beta cell stress and death are central components of type 1 diabetes (T1D) and also contribute in a decisive way to T2D (1, 2). Gertrude Stein once wrote in her poem “Sacred Emily” that a “rose is a rose is a rose is a rose.” But is “beta cell stress a beta cell stress a beta cell stress?” In other words, are all forms of beta cell stress the same and, more importantly, does it matter, as the outcome may be the same, namely relative or absolute reduction in insulin production?

Pancreatic beta cells are highly specialized endocrine cells that have as central tasks to sense circulating nutrients and respond to their circulating levels by releasing insulin in adequate amounts to assure their proper uptake and use by different organs; this maintains circulating levels of nutrients, such as glucose, inside narrow limits in spite of the wide variations in food intake observed in most mammalian species. Pancreatic beta cells in humans are very long lived, and our “beta cell patrimony” is probably established by early adulthood (3). These cells will thus need to cope with decades of varying insulin production – not a trivial task, considering that under stimulated conditions insulin synthesis increases 10-fold compared to basal level, approaching 50% of the total protein synthesis.

An important question is how beta cells react when exposed to mediators of autoimmune damage, such as pro-inflammatory cytokines released at the islet vicinity during the early [e.g., type 1 interferons (IFNs), such as IFNα] or late [e.g., interleukin-1β (IL1β) and interferon-γ (IFNγ)] stages of insulitis, or to saturated free fatty acids, such as palmitate, that contribute to metabolic stress in T2D (1, 2)? Available data suggest that beta cells trigger different adaptive responses that involve a decrease in its most differentiated functions, i.e., insulin synthesis and release, and the up-regulation of complex cellular responses, such as the unfolded protein response [UPR; (4)]. These adaptive responses are at least in part determined by the stress inducing them – for instance, beta cells exposed to pro-inflammatory cytokines trigger branches of the UPR that are different from the ones triggered in response to palmitate (2, 5, 6). These responses to diverse stresses will thus leave gene expression/molecular footprints that can be detected by omics techniques such as global RNA sequencing. Exam of these footprints may allow us to detect the nature of the beta cell stress causing them and, by comparing the molecular footprints induced by *in vitro* stresses with those present in beta cells isolated from patients affected by T1D, enable us to define the best experimental models to study the human disease. Furthermore, and of particular relevance for the discovery of novel therapies for T1D, comparisons of the different beta cells molecular footprints against large databases of cells exposed to different drugs, such as the recently updated Connectivity MAP database of cellular signatures, including > 1.3M profiles of human cells responses to chemical and genetic perturbations (7), can identify agents that antagonize particular gene signatures that may contribute to beta cell demise. Some of these agents, such as for instance the JAK inhibitor baricitinib, are already in use for other autoimmune diseases (8, 9) and can then be re-purposed for T1D therapy (10) (see below). We have recently published two comprehensive review articles focusing on beta cell fate in T1D (2, 11), and will focus here on the available studies characterizing the footprints left by immune or metabolic stresses on human beta cells.

In recent years RNA sequencing analysis has been done by us and others on human islets exposed to IL1β + IFNγ (12), IFNα (10) and palmitate (13) and of purified human beta cells or whole islets obtained from the pancreata of patients with T1D (14) or T2D (15); all these valuable datasets have been deposited on public access sites, such as the Gene Expression Omnibus repository (GEO). We have presently re-analyzed the most informative of these datasets, using the same pipeline [i.e., Salmon, GENCODE v31, DESeq2 (16–18)] to allow adequate comparisons between them, aiming to answer the following questions:

- How similar are the molecular footprints left on human islets by IL1β + IFNγ (12), IFNα (10) and palmitate (13)?
- Are these footprints representative of the patterns observed in beta cells obtained from patients affected by T1D?
- Can we obtain relevant indications for new therapies by mining these molecular footprints against available drug-induced footprints in other cell types?

## Methods

**Figure 1:**
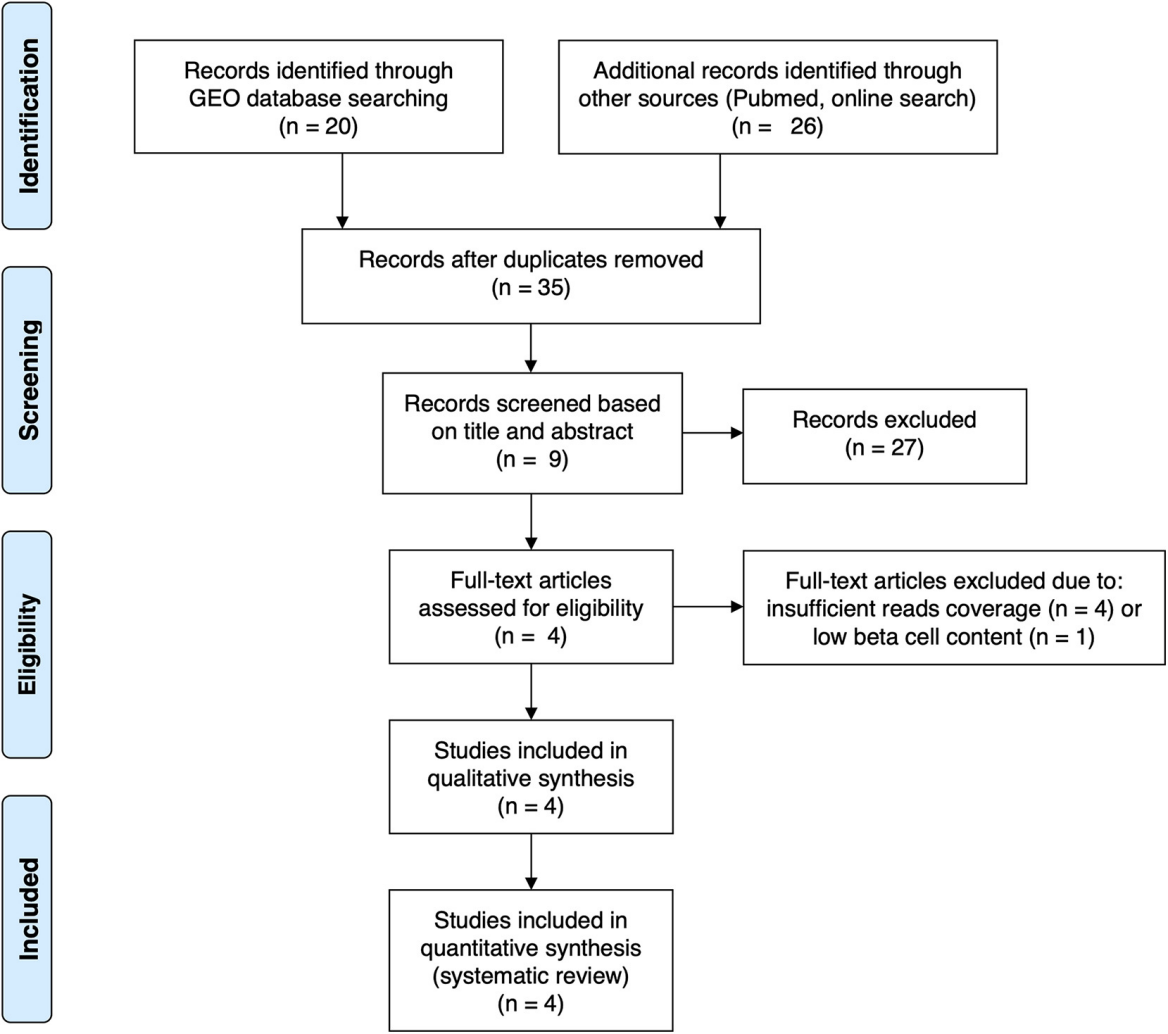
PRISMA flow diagram (19) describing the search strategy used to identify the analyzed studies.

For the present review and analysis we have selected available RNA-seq datasets of pancreatic human islets or FACS-purified human beta cells exposed to different pro-inflammatory stimuli (10, 12), metabolic stressors (13) or to the local environment present during T1D development (insulitis) (14) that are publicly available from the GEO repository (www.ncbi.nlm.nih.gov/geo). For the search we have used the following terms combinations: (1) “pancreatic endocrine cells” [All Fields] OR “pancreatic beta cells” [All Fields] OR “human islets” [All Fields] AND “type 1 diabetes” [All Fields] AND (“Homo sapiens” [Organism] AND “Expression profiling by high throughput sequencing”[Filter]); (2) “pancreatic endocrine cells” [All Fields] OR “pancreatic beta cells” [All Fields] OR “human islets” [All Fields] AND “cytokines” [All Fields] AND (“Homo sapiens” [Organism] AND “Expression profiling by high throughput sequencing” [Filter]); (3) “pancreatic endocrine cells” [All Fields] OR “pancreatic beta cells” [All Fields] OR “human islets” [All Fields] AND “palmitate” [All Fields] AND (“Homo sapiens” [Organism] AND “Expression profiling by high throughput sequencing” [Filter]). We also searched the Pubmed using the same criteria and mined online sources for unpublished data. Since the present analysis focus on beta cell transcript (isoforms) expression, we excluded articles having insufficient reads coverage (<20 million reads per sample, *n* = 3) and depleted of beta cells (<500 transcripts per million (TPM) of insulin, *n* = 1). The PRISM flow diagram (19) describing the search strategies is represented in Figure 1. Table 1 provides a detailed description of each dataset including their GEO reference number.

**Table 1:** Description of the datasets of pancreatic human islets and purified beta cells exposed to different stimuli and conditions presently evaluated.

**Treatment**	**Tissue**	**Donors**			**Duration**	***n***	**GEO**
		**Age (years)**	**Gender (M/F)**	**BMI**			
IL1β + IFNγ	Human islets	50.6 ± 22.8	2/3	25.1 ± 3.4	48 h	5	GSE108413
IFNα	Human islets	71.3 ± 17.7	1/5	26.4 ± 5.4	18 h	6	GSE133221
Palmitate	Human islets	55.2 ± 20.2	3/2	24.8 ± 1.6	48 h	5	GSE53949
**Condition**							
Control	FACS-purified beta cells	16.1 ± 5.8	8/4	NA	–	12	GSE121863
T1D	FACS-purified beta cells	19.7 ± 5.4	3/1	NA	3.1 ± 2.8 years	4	GSE121863

*GEO, Gene Expression Omnibus repository (https://www.ncbi.nlm.nih.gov/geo/); NA, non-available; M, male; F, female; BMI, body mass index. Data are mean ± standard deviation (10, 12–14)*.

After downloading the raw data, we used Salmon version 0.13.2 (16) to map the reads to the human reference transcriptome [GENCODE version 31 (GRCh38) (17)] using the quasi-alignment model. The transcript abundance is represented in Transcripts Per Million (TPM).

Differential analysis was performed using the R package DESeq2 version 1.24.0 (18). The estimated number of reads obtained from Salmon were used as input for DESeq2. Briefly, DESeq2 normalizes samples according to per-sample sequencing depth and accounting for intra-sample variability. Then, it fits data to a negative binomial generalized linear model (GLM) and calculates the Wald statistic. Finally, the raw *p*-values are corrected using the false discovery rate (FDR) for multiple testing by the Benjamini-Hochberg method. Transcripts with an FDR <0.05 were considered differentially modified.

To compare the different signatures present in each dataset, we performed Rank-Rank Hypergeometric Overlap (RRHO) mapping (20). For this goal, we have generated lists of transcripts ranked by their -log_10_ *p*-values from the differential expression analysis. In a RRHO map the hypergeometric *p*-value for enrichment of *k* overlapping genes is calculated for all possible threshold pairs for each condition, generating a matrix where the indices are the current rank in each condition. The log-transformed hypergeometric *p*-values are then plotted in a heatmap indicating the degree of statistically significant overlap between the two ranked lists on that position of the map. We have applied adjustment for multiple comparison using the Benjamini-Yekutieli correction.

To evaluate the similarities between datasets we used the R package FactoExtra version 1.0.6 (https://github.com/kassambara/factoextra) considering as (dis)similarity (distance) measure the Pearson correlation between samples (1 – correlation) using the 300 most variable transcripts (i.e., median absolute deviation). Next, hierarchical clustering was performed based on the average of the pairwise (dis)similarities (distances) between samples.

For functional enrichment analysis the R/ Bioconductor package ClusterProfiler version 3.12 (21) was used in combination with gene sets from the Molecular Signatures Database (22). All the transcripts presenting a TPM > 1 in at least half of the samples were considered as background and a Benjamini–Hochberg FDR threshold of 0.05 was defined as significant enrichment.

The top 150 up-regulated transcripts in the RNA-seq of FACS-purified beta cells from T1D individuals (14) were identified from the differential expression analysis. This list of transcripts was used to query the Connectivity Map dataset of L1000 cellular signatures, which has transcriptional responses of human cells to different chemical and genetic perturbations, using the CLUE platform (https://clue.io) (7). To identify compounds potentially reverting the effects induced by insulitis on beta cells we focused on perturbagens promoting signatures that were opposite (negative tau score) to our query list. Only perturbagens having a median tau score < −60 were considered for further evaluation.

## Results

### The Footprints of *in vitro* Cytokine, but Not Palmitate, Exposure Are Similar to the Ones Observed in Beta Cells From T1D Patients

**Figure 2:**
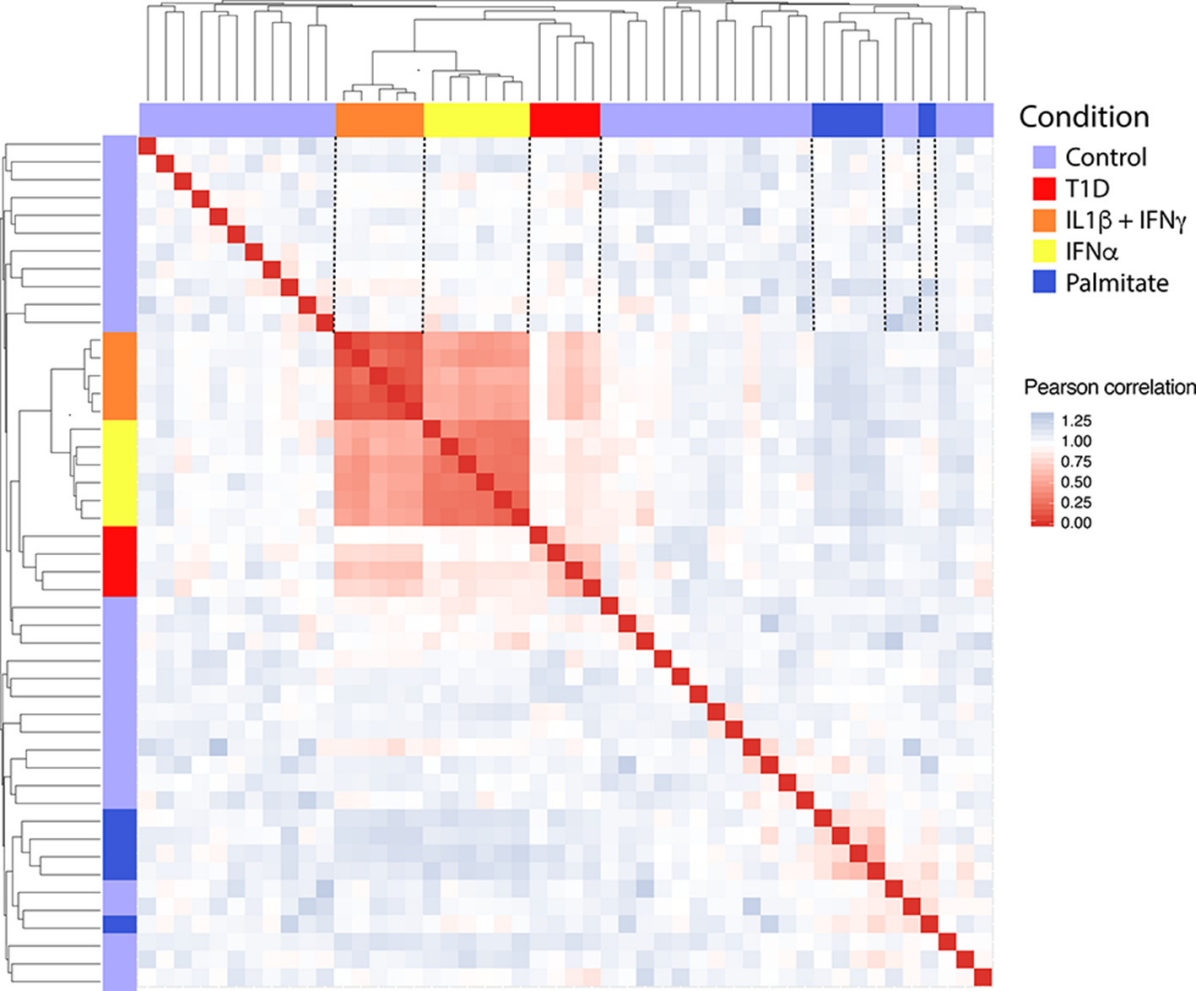
Correlation between the transcripts expressed in human islets exposed to IL1β + IFNγ, IFNα or palmitate and the transcripts expressed in human beta cells of individuals affected by T1D. Data were obtained by RNA sequencing (10, 12–14). The Pearson correlation (1 – correlation) was used to evaluate the (dis)similarities (distance) among the 300 most variable transcripts in the RNA-seq datasets. Red squares represent a positive correlation (similarity), blue squares a negative correlation (dissimilarity) and white squares an absence of correlation between each pair of observations. Next, the hierarchical clustering was performed considering the average of the dissimilarity (distance) between samples. The resulting dendrogram is shown in the upper and lateral part of the matrix.

In order to obtain *in vitro* inflammatory and metabolic footprints of beta cells, we analyzed previously generated RNA-sequencing of pancreatic human islets exposed to pro-inflammatory cytokines [IL1β + IFNγ (12) or IFNα (10)] or to a metabolic stressor [palmitate (13)] (Figure 1). Furthermore, recent advances in techniques to purify beta cells and the establishment of collaborative networks between different research groups have allowed for the first time the generation of RNA-sequencing data of human beta cells from T1D individuals (14). This database offers a unique opportunity to validate the *in vitro* models by comparing them against the *in vivo* situation present during T1D development. For this purpose, we first evaluated the similarities between the signatures of transcripts induced by the different stimuli (inflammatory and metabolic) in pancreatic islets and by the local environment of insulitis that beta cells are exposed to in T1D (Figure 2). The analysis was performed at the transcript (isoform) level, since we have previously observed that exposure of beta cell to pro-inflammatory cytokines promotes major changes in alternative splicing (AS), leading to a high number of different splicing events (10, 23–25). This is particularly relevant since AS is a cell-type- and context-dependent mechanism. In line with this, several RNA-binding proteins that regulate gene splicing are significantly modified in beta cells isolated from individuals affected by T1D (Supplementary Table 1). This analysis indicated that the pro-inflammatory cytokines trigger a profile of transcripts that generate clusters of similar samples (positive Pearson correlation), while the metabolic stressor palmitate generates different groups of transcripts that cluster separately and with an opposite profile as compared to cytokines (negative Pearson correlation) (Figure 2). Interestingly, the samples obtained from T1D individuals (indicated by red color) clustered together with the two *in vitro* models of pancreatic islet inflammation; this similarity was slightly higher (represented by darker red color boxes) with the signature of the pro-inflammatory cytokines IL1β + IFNγ (orange) than with IFNα (yellow) (Figure 2). This is probably due to the fact that the beta cell samples were obtained from four patients 5 months, 2, 3, and 7 years after diagnosis of T1D, a period when a full adaptive immune response against the beta cells is in place, including exposure of islets to the cytokines IL1β + IFNγ, while IFNα may play a more relevant role during the early and more “innate-immunity related” phases of the disease (26).

**Figure 3:**
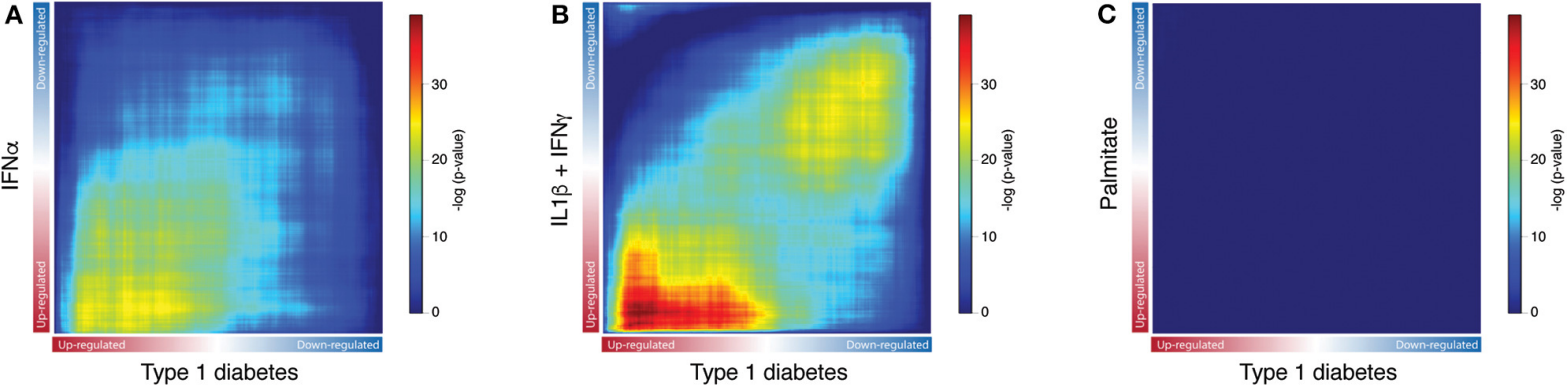
Exposure of human islets to pro-inflammatory cytokines, but not to palmitate, induce a similar transcriptomic profile as observed in islets isolated from patients affected by type 1 diabetes. **(A–C)** Rank-Rank Hypergeometric Overlap (RRHO) map comparing the transcriptional expression profile of human islets exposed to IFNα (**A**), IL1β + IFNγ (**B**) or palmitate (**C**) to the one present in primary beta cells from individuals affected by T1D, as identified by RNA-seq. Ranked lists of transcripts based on the -log_10_ *p*-values from the differential expression analysis of human islets exposed to IFNα (**A**), IL1β + IFNγ (**B**) or palmitate (**C**) were compared to a similarly ranked-list from beta cells obtained from patients with T1D.

Next, to identify the transcripts that are analogously modulated in beta cells from individuals affected by T1D and in pancreatic islets exposed to the different stressful stimuli, we performed a Rank-Rank hypergeometric overlap (RRHO) analysis (20) which evaluates the (dis)similarities between two ranked lists. For this comparison, ranked lists of transcripts based on the -log_10_ *p*-values from the differential expression analysis (T1D or stimuli vs. controls) were generated. The RRHO mapping demonstrated a significant intersection of similarly up-regulated transcripts in T1D beta cells and in human islets exposed to both IFNα (Figure 3A) and IL1β + IFNγ (Figure 3B). In agreement with the distance matrix findings (Figure 2), the significance of this intersection was more pronounced for the late cytokines, i.e., IL1β + IFNγ (Figure 3B), particularly related to down-regulated genes. This concordance in down-regulated genes may be due to the fact that IL1β + IFNγ but not IFNα, trigger a more severe beta cell stress, eventually leading to apoptosis (10, 23, 27). On the other hand, there was no statistically significant correlation between the T1D beta cells signature and the one induced by the metabolic stressor palmitate in human islets (Figure 3C). In line with this, we have previously shown that there is no clear correlation between human islets exposed to IFNα (10) or to IL1β + IFNγ (2) and human islets obtained from patients affected by type 2 diabetes.

**Figure 4:**
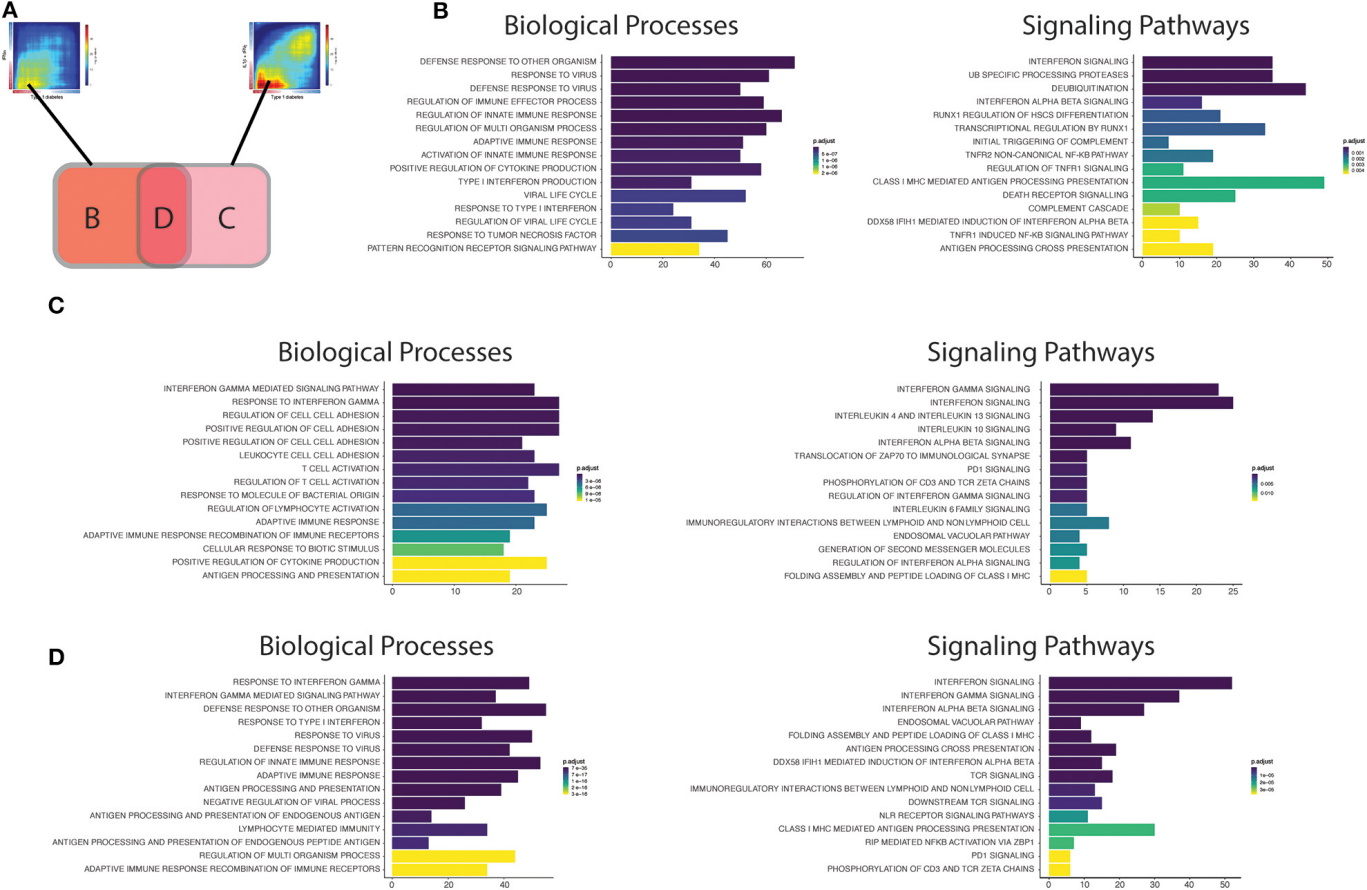
Functional analysis of the transcripts overlapping beta cell datasets from T1D patients' islets or cytokine-exposed human islets. **(A)** First, the up-regulated transcripts present in the intersection of RRHO maps comparing the T1D signature in beta cells against the IFNα (left) and IL1β + IFNγ (right) signatures in human islets were identified. Next, these transcripts were divided in three different groups: (**B**) transcripts only present in the intersection between T1D vs. IFNα, (**C**) transcripts only present in the intersection between T1D vs IL1β + IFNγ, (**D**) transcripts present in the intersection of all the three datasets (T1D, IFNα, IL1β + IFNγ). (**B–D)** Biological processes (Gene Ontology) and signaling pathways (Reactome) enriched among the overlapping regions of the RRHO maps described above [**(B)** only T1D vs IFNα; (**C)** only T1D vs. IL1β + IFNγ; **(D)** T1D, IFNα and IL1β + IFNγ]. The y axis shows the 15 most significant genesets, while the x axis represents the total number of transcripts identified in each geneset.

To gain further insight into the biological processes and pathways triggered at the different stages of T1D development, we next performed enrichment analysis of the up-regulated transcripts present in the areas of significant intersection between the RNA-seq datasets from islets obtained from patients affected by T1D as compared to cytokine-treated human islets (Figure 4). Figure 4A outlines the three intersection areas evaluated, while Figure 4B shows transcripts up-regulated only in the intersection between the INFα and T1D datasets, reflecting most likely the early changes induced in beta cells during the evolution of diabetes. Figure 4C shows transcripts up-regulated only in the intersection between the IL1β + INFγ and T1D datasets, probably mirroring the changes present at later stage of T1D progression. Finally, Figure 4D shows transcripts up-regulated in the intersection between all datasets (INFα, IL1β + IFNγ, and T1D), which may represent alterations common to different phases of the disease. These putative early and late changes observed in the islets in the course of T1D are discussed in more detail below.

### Early Pancreatic Islets Changes

The biological processes (Gene Ontology) regulated by the transcripts present in area **B** (Figure 4B; only T1D vs. IFNα) recapitulate well the initial events putatively present during T1D development (26). In brief, innate immune responses are probably triggered after activation of pattern recognition receptors (PRRs) (including Toll-like receptors (TLRs), and RIG-I-like receptors) by endogenous “danger signals” or exogenous ligands, such as nucleic acids (e.g., double stranded RNA) produced during viral infections, which are putative environmental factors associated with T1D (28). The activation of PRRs on beta cells promotes an increased local production of antiviral type I interferons, such as IFNα, by the beta cells themselves and by other cells present in the islets, and the recruitment of immune cells to the pancreatic islets. In support to this model, rare variants in the RIG-I-like receptor *MDA5* that lead to decreased function are associated with protection against T1D (29). In contrast, a genetic variant of *MDA5* that leads to a partial gain-of-function significantly increases the risk for T1D (30). In both cases, the impact on T1D development is dependent on, respectively, a decreased or an exacerbated production of type I IFNs. These locally produced cytokines activate several signaling pathways that include key pathways observed in early human T1D, such as MHC class I antigen presentation (31) and beta cell death (26) (Figure 4B). We have recently demonstrated that IFNα up-regulates different mechanisms involved in post-transcriptional regulation of gene expression, especially alternative splicing (AS) (10) and endoplasmic reticulum (ER) stress (10, 32), which can potentially generate beta cells neoantigens. This, combined with the overexpression of HLA class I, may facilitate auto-immunity progression by activation of autoreactive T-cells that may have escaped thymic selection (11). Of note, a similar phenomenon has been observed for the pro-inflammatory cytokines IL1β + IFNγ which increase the expression of an isoform of secretogranin V, SCG55-009, that is recognized by auto-reactive CD8^+^ T-cells present in the pancreas of T1D individuals (12). It has also been described that beta cells undergoing endoplasmic reticulum stress can modify the insulin translation initiation site, producing a highly immunogenic polypeptide capable of activating T-cells from T1D individuals with the potential for killing human beta cells (33).

Macrophages are among the main immune cell populations present in the human pancreas (34), and their density is increased in the vicinity of the pancreatic islets (<20 μM) of individuals with recent-onset T1D in comparison with non-diabetic individuals (34). This is compatible with findings from different animal models showing that macrophages are among the first activated immune cells present in the pancreas in autoimmune diabetes (35, 36) and the main cell type responsible for the TNF production in pancreatic islets (37). Furthermore, we identified “activation of TNF receptor subunits” and its main downstream transcription factor nuclear factor-κB (NF- κB) as important pathways activated in early human T1D (Figure 4B). This is particularly relevant, since differently from other cell types in which NF-κB has mainly a pro-survival role (38), NF-κB activation in beta cells is mostly pro-apoptotic (39).

Finally, pathways involved in the regulation of protein ubiquitination were also overrepresented in our analysis of common effects observed in early T1D and following human islet exposure to IFNα (Figure 4B). The signal transduction downstream of the PRRs relies on their association with specific adaptors, which in many cases require ubiquitin-specific modifications to became active (40). Ubiquitination is counteracted by deubiquitinases, a group of enzymes that remove these modifications and thus provide a negative feedback on the signaling cascade (41). Interestingly, single-nucleotide polymorphisms (SNPs) that decrease activity of the ubiquitin-modifying enzyme *TNFAIP3* (A20) (A20 provides a negative feedback on NF-κB activation by stimulating degradation of some of its components) are associated with a higher risk for development of T1D and other autoimmune diseases (42). Moreover, rare mutations leading to TNFAIP3 loss-of-function cause a systemic autoinflammatory disease (43). The affected individuals present a type I interferon (IFN) signature which correlates with the disease activity and predicts their response to treatment with janus kinase (JAK) inhibitors (JAK1 is a key kinase for type I interferon signaling) (44). In agreement with this, we have shown that the JAK 1 and 2 inhibitors ruxolitinib (45) and baricitinib (10) prevent IFNα-induced MHC class I and chemokine up-regulation in human islets, besides inhibiting IFNα + IL1β-induced beta cell apoptosis; another drug from this family was shown to prevent diabetes in NOD mice (46). Baricitinib is already in clinical use for other autoimmune diseases (8, 9) and may be eventually re-purposed for the early therapy of T1D. This demonstrates the utility of beta cell signature characterizations for the identification of new therapeutic targets in T1D (see below).

### Advanced Pancreatic Islets Changes

In the case the local pro-inflammatory environment described above is maintained, the increased homing of different immune cells to the pancreatic islets promotes the transition to a scenario dominated by adaptive immune responses. The intersection area **C** may be representative of the findings observed during these late stages of insulitis (Figure 4C). The biological processes (GO) are now enriched in IFNγ responses, reflecting the increased number of T-cells present in the islets. A critical step for the immune cells to reach the inflamed tissue is their adhesion and crawling on the endothelium (47). In line with this, several processes (GO) involved in cell adhesion are induced in pancreatic islets from T1D individuals (Figure 4C). During this process, activated T-cells expressing high-affinity integrins bind to the endothelial cells via cellular adhesion molecules (CAMs) (48). Deficiency of the vascular adhesion molecule adhesion intercellular adhesion molecule 1 (ICAM1) (49) or its receptor (50), lymphocyte function-associated antigen 1 (LFA1), prevents the development/progression of autoimmune diabetes in NOD mice. Of interest, a genetic risk variant associated to T1D (rs657152) (51) is also associated with the circulating levels of soluble ICAM1 (52).

Other potentially important pathways identified by the present analysis were the “Immunoregulatory interactions between lymphoid and non-lymphoid cells” and “PD1 signaling” (Figure 4C). We have recently shown that during insulitis, in addition to pro-inflammatory stimuli, beta cells also express immune checkpoint proteins, including programmed death-ligand 1 (PDL1) and HLA-E, possibly in an attempt to down-regulate the immune responses and thus avoid further tissue damage (10, 53). In line with these observations, individuals receiving immunotherapy based on PDL1/PD1 blockers for cancer treatment have a higher risk of developing T1D (54) and other autoimmune diseases (55). The induction of these checkpoint proteins in human beta cells is mainly mediated by type I and II IFNs under the control of the transcription factor interferon regulatory factor 1 (IRF1) (53). The protective role for these co-inhibitory molecules in beta cells is reinforced by the facts that beta cells surviving the immune assault in NOD mice express high levels of PDL1 (56), that transgenic PDL1 overexpression in beta cells decreases diabetes prevalence in NOD mice (57), and that that both PDL1 (53) and HLA-E (10) are absent in pancreatic islets from T1D individuals depleted of insulin.

Another group of immunomodulatory molecules presenting activation of their signaling pathways were anti-inflammatory cytokines, including interleukin-10 (IL10) and interleukin 4/13 (IL4/13) (Figure 4D). In line with this, systemic delivery of IL10 via adenovirus-associated gene therapy prevented diabetes recurrence after syngeneic islets transplantation in NOD mice (58). In the same animal model, oral administration of a probiotic (*Lactococcus lactis*) expressing IL10 and the autoantigen GAD65, in combination with low dose of anti-CD3, reversed autoimmune diabetes (59). The second class of cytokines include IL4 and IL13, which exerts their actions through three different combinations of shared receptors (60). These cytokines can trigger phenotypes that range from allergy, including asthma, to anti-helminthic responses. Interestingly, helminthic infections are associated with protection against immune-mediated diseases, such as T1D (61). The systemic administration of IL4 (62) or IL13 (63) was shown to prevent the development of diabetes in NOD mice. This effect is at least in part mediated via their direct action on beta cells, since beta cells express all the required IL4/13 receptor subunits (64) and their *in vitro* exposure to IL4 or IL13 protects them against pro-inflammatory cytokine-induced apoptosis (64, 65). This cytoprotection is associated with the activation of signal transducer and activator of transcription 6 (STAT6) in beta cells, leading to the up-regulation of anti-apoptotic proteins such as myeloid leukemia-1 (MCL-1) and B cell lymphoma-extra large (BCL-XL) (65).

Finally, analysis of the biological processes and the signaling pathways controlled by genes commonly up-regulated in all the three datasets (IFNα, IL1β + IFNγ and T1D) (Figure 4D) indicate that they summarize many findings present at the different stages of the disease, including regulation of antiviral responses, responses to type I (IFNα/β) and II (IFNγ) interferons, MHC class I antigen presentation, lymphocyte activation, interaction between lymphoid and non-lymphoid cells and PD1 signaling. This suggest that signatures of both innate and adaptive immunity remain present in the islets as the disease evolves, which is supported by histological and RNA-seq findings in whole islets from T1D patients (66, 67). Analysis of transcripts that are only modified in beta cells of T1D individuals, but not after exposure of these cells to cytokines (Supplementary Figure 1A), indicates pathways that are less dependent on these inflammatory mediators (Supplementary Figure 1B). Changes in genes involved in digestion and absorption were upregulated in samples from T1D only, and among these genes we identified CTRB1 and CTRB2. Polymorphisms in these genes have been associated to higher risk for T1D development (68, 69), and mild hyperglycemia induces their upregulation and that of other genes identified in the same pathway (70). There was also activation of matrix metalloproteinases, which is a potentially relevant mechanism in T1D since matrix metalloproteinases can cleave membrane-bound PDL1 present on the cell surface and thus regulate T-cell responses (71).

### Mining Beta Cell Molecular Footprints for Drug Re-purposing in T1D

**Figure 5:**
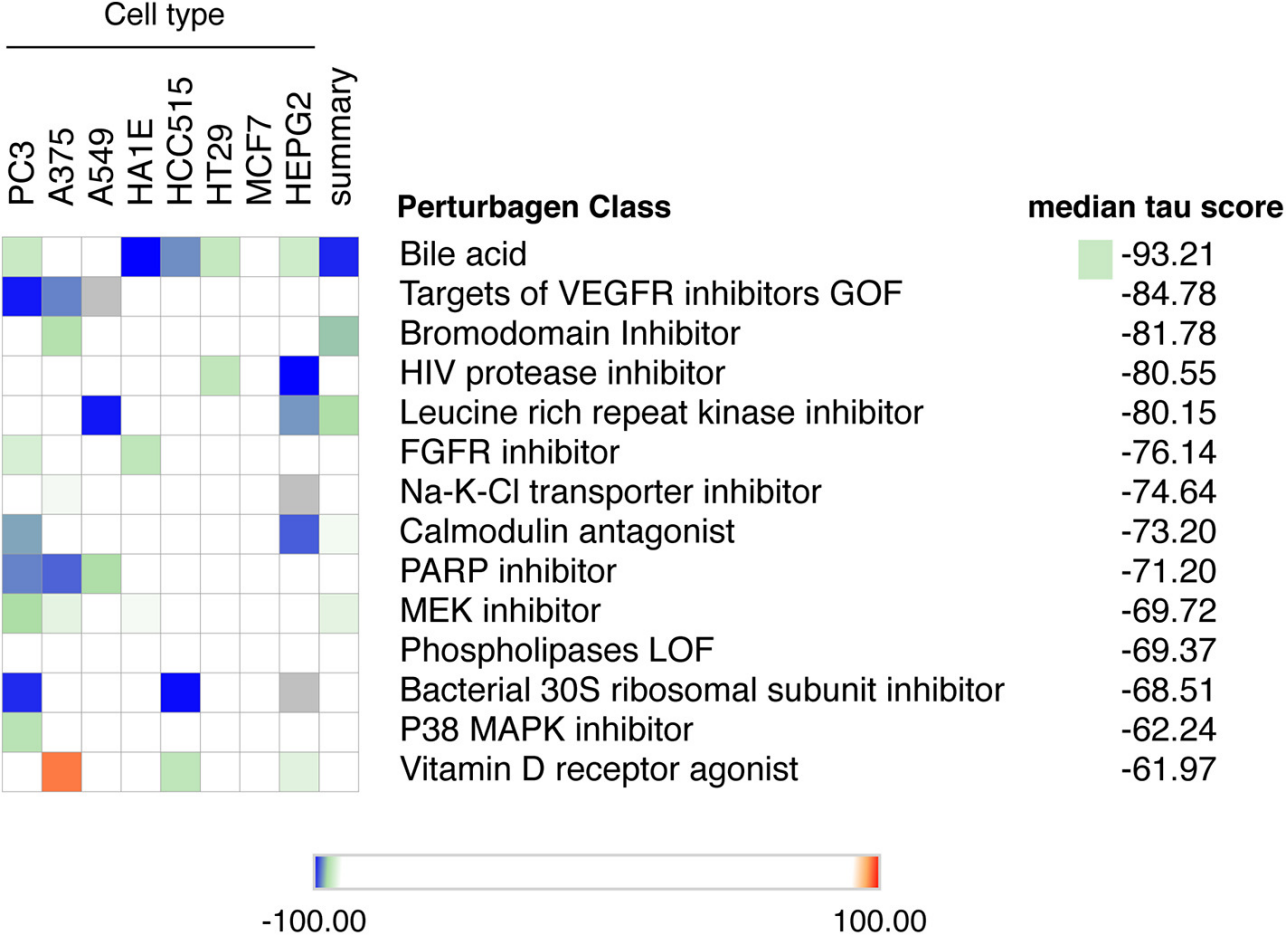
Mining the beta cell signature in T1D to identify potential new therapeutic targets. The top 150 up-regulated transcripts identified in the RNA-seq of beta cells of T1D individuals (14) were used to query the Connectivity Map database of cellular signatures (7). The top Connectivity Map classes of perturbagens that promote an opposite signature (negative tau scores) to the one present in beta cells of T1D individuals are represented.

Up to now there is no treatment available to prevent the development of T1D in individuals at risk. This, and the worldwide increase in T1D incidence observed in recent decades (72, 73), makes T1D a major area of interest for drug discovery. We have presently mined the RNA-sequencing of FACS-purified beta of T1D individuals (14) using the recently updated version of the Connectivity Map (CMap) database (7). To avoid potential off-target findings caused by focusing on individual compounds in the analysis, we focused instead on classes of drugs that promote an opposite signature to the one present in beta cells of T1D individuals. We identified several classes of drugs/compounds that could potentially revert the inflammatory signatures present in beta cells during T1D, including bile acids, bromodomain inhibitors, leucine-rich repeat kinase (LRRK) inhibitors and vitamin D receptor agonists (Figure 5).

Among the top classes of compounds identified were bile acids. This is an interesting finding, since TUDCA - a conjugated bile acid with chaperone properties - has been shown to prevent autoimmune diabetes in NOD mice (74). This protection was mediated via the restoration of a defective unfolded protein response (UPR) observed in beta cells during insulitis. *In vitro* studies demonstrated that TUDCA also inhibits IFNα-induced ER stress and its subsequent UPR activation in human beta cells (75). Finally, there is an ongoing phase 2 clinical trial evaluating the potential translational impact of these findings in individuals with recent-onset T1D (ClinicalTrials.gov Identifier: NCT02218619).

Another class of compounds identified were bromodomain inhibitors. The bromodomain (BRD) proteins are “readers” of histone acetylation that, associated with other components of chromatin-remodeling complexes, promote transcriptional activation. Bromodomain and extra-terminal domain (BET) proteins are the most studied members of the BRD family. Inhibitors of BET proteins have protective effects in different animal models of autoimmunity (76, 77), including in the NOD mouse model of autoimmune diabetes (78). We have recently demonstrated that two bromodomain inhibitors, namely JQ1 and I-BET-151, partially prevent the deleterious effects of IFNα on human beta cells (10).

We also identified leucine-rich repeat kinase inhibitors (LRRK) as a potential perturbagen in T1D (Figure 5). The currently available LRRK inhibitors mainly target LRRK2. The LRRK2 protein has two domains with catalytic activity; a GTPase domain of the Ras of complex (ROC) protein family, and a kinase domain of the tyrosine kinase like (TKL) family (79). Genome-wide association studies have linked kinase-activating mutations in LRRK2 with an increased risk for both Parkinson disease (80) and inflammatory bowel diseases (IBD) (81). *In vivo* and *in vitro* studies indicate that these LRRK2 risk variants act mainly by promoting exacerbated responses to pro-inflammatory stimuli (82–84). In line with this, LRRK2 inhibition prevented microglial inflammatory responses triggered by TLR activation (85). Of concern, systemic LRRK2 knockout (KO) prevented the phosphorylation of Rab10, a crucial step for insulin-mediated glucose transporter type 4 (GLUT4) translocation to cell surface (86). GLUT4 is a key glucose transporter in peripheral tissues, and this inhibitory impact on its cellular localization may lead to insulin resistance. This highlights the importance of reviewing the whole body of evidence before selecting the best potential therapeutic targets.

Several studies have demonstrated the role of vitamin D and its analogs as anti-inflammatory and immunomodulatory agents. In line with this, exposure of human pancreatic islets to the active form of vitamin D, calcitriol, increases expression of the protective candidate gene *TNFAIP3* and reduces pro-inflammatory cytokine (IL1β + TNF + IFNγ)-induced MHC class I expression, IL6 production and nitric oxide synthesis (87, 88). *In vivo* studies on NOD mice support these findings, with a decrease in insulitis and diabetes prevalence observed when calcitriol was administrated early in life (89). Furthermore, two large birth cohorts of genetically at risk individuals demonstrated that lower levels of 25(OH)-vitamin D in early infancy are associated with a higher incidence of islet autoimmunity (90, 91) and T1D development (90). This effect was modified by polymorphisms in the vitamin D receptor (VDR) (91). However, data from clinical trials using vitamin D and its analogies in humans have provided conflicting results. This may be caused by different factors: (1) the genetic background of the affected individuals (such as genetic variants in the candidate gene *TNFAIP3* and polymorphisms in the VDR) may modify their responses to vitamin D; (2) insufficient doses of vitamin D to modulate the immune system are often used, due to concerns regarding hypercalcemia; (3) too late introduction of the treatment; (4) the need to use vitamin D analogs in combination with other agents to address the complexity of T1D pathogenesis.

## Discussion

Despite the continued work performed by the research community there is still no treatment capable of preventing T1D development in individuals at risk or reverting disease after its outbreak. At the very best, use of anti-CD3, a class of drugs targeting exclusively the immune system, delayed by around 2 years the progression to T1D in individuals at high-risk for the disease (92). A similarly transient benefit on beta cell function was observed when treating patients with clinical T1D using anti-CD3 (93) or anti-CD20 (94).

In order to better understand and treat T1D, we may need to move on from an immune cell-centered view of the disease to a scenario that considers the disease as the product of a dynamic interaction between the killing immune cells and the target beta cells (2, 11, 26). To add information on the impact of insulitis on the target beta cells, we have presently characterized the human beta cell responses during T1D and after exposure to different immune-mediated stimuli. We observed that the *in vivo* beta cell responses can be closely recapitulated using an *in vitro* system biology approach that combines exposure to cytokines putatively present at different stages of the disease and high coverage RNA-sequencing. This may be further improved by the parallel evaluation of cytokine-induced changes in human beta cells protein expression (10, 95, 96) and chromatin status (10, 95). The validation of these models is an important finding, as the access to high quality beta cells from patients affected by T1D for multi-omics analysis is extremely difficult. Finally, the present integrated analysis identified important biological processes and signaling pathways activated during T1D progression, leading to the identification of potentially new therapeutic targets to be considered for future clinical trials.

Recent studies have suggested a role for aging and senescence-associated secretory profile (SASP) in beta cell disfunction and death in experimental models for both T1D and T2D (97, 98). We have searched for eventual changes in SASP-related genes (i.e., ARNTL, CDKN1A, ICAM1, ID2, LIMS1, MAP2K1, MAPK14, MIF, PRKCD, SERPINE1, TBX2, ULK3, ZMIZ1 etc.) in the present RNA-seq of beta cells from T1D individuals, but did not observe significant changes in any of these transcripts as compared to non-diabetic donors (data not shown).

Weakness of the present study, which may hamper extrapolations to most T1D patients, include the limited number of samples analyzed (there were only 4 preparations from T1D patients available in the literature that reached the proposed criteria (see Methods) for inclusion in our analysis) and the disparate age of islet/beta cell donors when comparing for instance T1D patients (mean age 19.7 years) and IFNα-treated islets (mean age 71.3 years) (Table 1). It is remarkable that, in spite of these limitations, there remained major and consistent similarities between the gene expression observed in beta cells from different patients affected by T1D and the gene expression present in human islets exposed to pro-inflammatory cytokines.

To further expand the present information and better define the role of each cell type present in the islets of Langerhans or in its vicinity during insulitis, single cells analysis will be required (99); up to now, however, this has been technically challenging due to the very limited number of beta cells retrieved from diabetic individuals (100). This approach has been already performed in another autoimmune disease, systemic lupus erythematosus, in which single cell analysis of lupus nephritis identified cell specific signatures that led to the recognition of key signaling pathways suitable for specific therapeutic targeting (101).

The recent technical and scientific advances allowing the generation of beta cells derived from inducible pluripotent stem cells (iPSC) that show similar responses to pro-inflammatory cytokines as adult human beta cells (102), opens the possibility to differentiate beta cells from individuals with specific genetic variants that modulate the different stages of insulitis (2). Notably, these iPSC-derived cells could also be used to generate organoids simulating the pancreas environment, which would allow co-culture with relevant immune cells to better define beta cell-immune system crosstalk in the context of specific genetic backgrounds, and then to use this system for drug screening.

T1D is a complex disease, and a sustained therapeutic response will only be achieved by combining compounds that contribute to “re-educate” the immune system and to protect/regenerate beta cells. An interesting approach to be pursued at the beta cell level would be to down-regulate HLA class I antigen presentation while increasing signaling of the immune check-point proteins (PDL1 and HLA-E); this may be feasible, as suggested by the observation that inhibiting the transcription factor STAT2 decreases HLA class I expression while preserving/increasing PDL1 expression (53). Importantly, these interventions should be safe and started as early as possible to prevent irreversible loss of functional beta cell mass.

## Data Availability Statement

Publicly available datasets were analyzed in this study. This data can be found at: Gene Expression Omnibus (GEO) repository (https://www.ncbi.nlm.nih.gov/geo/) under the accession numbers: GSE108413, GSE133221, GSE53949, GSE121863, GSE121863.

## Author Contributions

MC and DE conceived, designed, supervised the study, wrote the manuscript, and all authors revised it. MC retrieved data, performed analysis, as such have full access to all the data in the study, take responsibility for the integrity of the data, and the accuracy of the data analysis. MC and FS performed bioinformatic analyses. All authors contributed to the article and approved the submitted version.

## Conflict of Interest

The authors declare that the research was conducted in the absence of any commercial or financial relationships that could be construed as a potential conflict of interest.
