# Supplementary material for: Molecular Footprints of the Immune Assault on Pancreatic Beta Cells in Type 1 Diabetes
Source: Front Endocrinol (Lausanne). 2020 Sep 15;11:568446. doi: 10.3389/fendo.2020.568446 (PMC7522353; doi:10.3389/fendo.2020.568446)
Supplement: Supplementary file 1 [file Data_Sheet_1.PDF]

Gene ID	Transcript Name	Gene Name	Log2 FC	FDR
ENST00000373993	A1CF-202	A1CF	-8.25	0.04
ENST00000290585	CELF3-202	CELF3	-1.01	0.02
ENST00000382401	CPEB2-204	CPEB2	-23.96	0.00
ENST00000517610	ESRP1-206	ESRP1	-9.07	0.00
ENST00000523347	ESRP1-211		-6.83	0.00
ENST00000370475	FMR1-205	FMR1	4.62	0.04
ENST00000635647	PTBP1-221	PTBP1	4.48	0.01
ENST00000463509	RBFOX2-211	RBFOX2	-1.41	0.03
ENST00000651280	SART3-217	SART3	-22.01	0.00
ENST00000339436	SRSF3-201	SRSF3	6.87	0.00
ENST00000621715	TARDBP-225	TARDBP	-7.59	0.01
ENST00000474699	TIA1-209	TIA1	-3.06	0.03
ENST00000393514	ZC3H14-205	ZC3H14	-7.95	0.02

Supplementary Table 1. Isoforms of RNA binding proteins differentially regulated in beta cells during T1D. The table represents the transcripts of RNA-binding proteins that are significantly modified at mRNA level (False discovery rate (FDR) < 0.05) in RNA-sequencing of beta cells obtained from T1D individuals in comparison to healthy individuals (1). FC: fold-change.

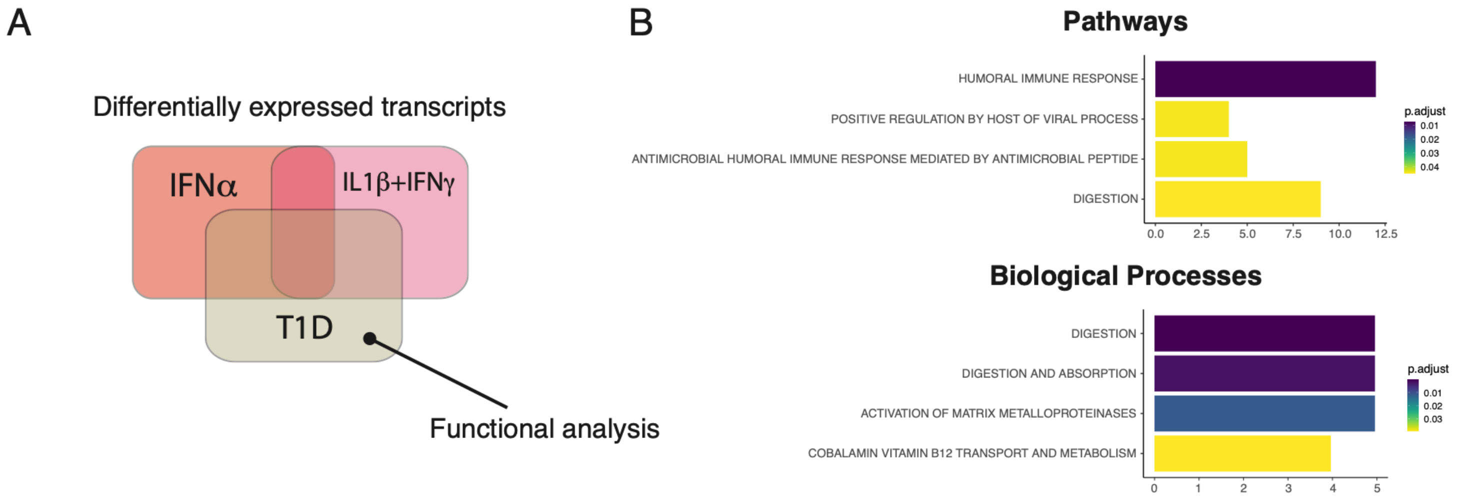

Supplementary Figure 1. Functional analysis of transcripts differentially expressed in purified beta cells of T1D individuals but not in cytokine-exposed human islets. A. Intersection of differentially expressed transcripts between purified beta cells of T1D individuals (1) and human islets exposed to IFNα (2) or IL1β + IFNγ (3) was performed. Only transcripts significantly modified by T1D but not by cytokines were selected for functional enrichment analysis. Transcripts were considered significantly modified if presenting a |FC| > 1.5 and a FDR < 0.05 in DESeq2 results. **B.** Signaling pathways (Reactome) and biological processes (GO) overrepresented among transcripts upregulated only in beta cells from T1D individuals.

References:

1. Russell MA, Redick SD, Blodgett DM, Richardson SJ, Leete P, Krogvold L, Dahl-Jorgensen K, Bottino R, Brissova M, Spaeth JM, Babon JAB, Haliyur R, Powers AC, Yang C, Kent SC, Derr AG, Kucukural A, Garber MG, Morgan NG, Harlan DM. HLA Class II Antigen Processing and Presentation Pathway Components Demonstrated by Transcriptome and Protein Analyses of Islet beta-Cells From Donors With Type 1 Diabetes. Diabetes. 2019;68(5):988-1001. Epub 2019/03/06. doi: 10.2337/db18-0686. PubMed PMID: 30833470; PMCID: PMC6477908.
2. Colli ML, Ramos-Rodriguez M, Nakayasu ES, Alvelos MI, Lopes M, Hill JLE, Turatsinze JV, Coomans de Brachene A, Russell MA, Raurell-Vila H, Castela A, Juan-Mateu J, Webb-Robertson BM, Krogvold L, Dahl-Jorgensen K, Marselli L, Marchetti P, Richardson SJ, Morgan NG, Metz TO, Pasquali L, Eizirik DL. An integrated multi-omics approach identifies the landscape of interferon-alpha-mediated responses of human pancreatic beta cells. Nat Commun. 2020;11(1):2584. Epub 2020/05/24. doi: 10.1038/s41467-020-16327-0. PubMed PMID: 32444635; PMCID: PMC7244579.
3. Gonzalez-Duque S, Azoury ME, Colli ML, Afonso G, Turatsinze JV, Nigi L, Lalanne AI, Sebastiani G, Carre A, Pinto S, Culina S, Corcos N, Bugliani M, Marchetti P, Armanet M, Diedisheim M, Kyewski B, Steinmetz LM, Buus S, You S, Dubois-Laforgue D, Larger E, Beressi JP, Bruno G, Dotta F, Scharfmann R, Eizirik DL, Verdier Y, Vinh J, Mallone R. Conventional and Neo-antigenic Peptides Presented by beta Cells Are Targeted by Circulating Naive CD8+ T Cells in Type 1 Diabetic and Healthy Donors. Cell Metab. 2018;28(6):946-60 e6. Epub 2018/08/07. doi: 10.1016/j.cmet.2018.07.007. PubMed PMID: 30078552.
